# Surgical Strategies for Native Esophagus Preservation in Long-Gap Esophageal Atresia: A 20-Year Population-Based Study

**DOI:** 10.3390/jcm15176895

**Published:** 2026-09-06

**Authors:** Maja Milickovic, Dragana Vujovic, Petar Rasic, Marija Stevic, Svetlana Vrzic-Petronijevic, Jelena Rakocevic, Blagoje Grujic, Aleksandar Vlahovic, Sinisa Ducic, Sanja Sindjic-Antunovic

**Affiliations:** 1 Faculty of Medicine, University of Belgrade, 11000 Belgrade, Serbia; maja.milickovic@imd.org.rs (M.M.); dragana.vujovic@udk.bg.ac.rs (D.V.); marija.stevic@med.bg.ac.rs (M.S.); svetlana.vrzic-petronijevic@med.bg.ac.rs (S.V.-P.); blagoje.grujic@imd.org.rs (B.G.); aleksandar.vlahovic@imd.org.rs (A.V.); sinisa.ducic@udk.bg.ac.rs (S.D.); 2 Department of Abdominal Surgery, Mother and Child Health Care Institute of Serbia “Dr. Vukan Cupic”, 11000 Belgrade, Serbia; 3 Department of Neonatal Surgery, University Children’s Hospital, 11000 Belgrade, Serbia; 4 Department of Anesthesiology, Reanimatology and Intensive Care, University Children’s Hospital, 11000 Belgrade, Serbia; 5 Clinic for Obstetrics and Gynecology, University Clinical Center of Serbia, 11000 Belgrade, Serbia; 6 Institute of Histology and Embryology, Faculty of Medicine, University of Belgrade, 11000 Belgrade, Serbia; jelena.rakocevic@med.bg.ac.rs; 7 Department of Neonatal Surgery, Mother and Child Health Care Institute of Serbia “Dr. Vukan Cupic”, 11000 Belgrade, Serbia; 8 Department of Plastic and Reconstructive Surgery and Burns, Mother and Child Health Care Institute of Serbia “Dr. Vukan Cupic”, 11000 Belgrade, Serbia; 9 Department of Orthopedics and Traumatology, University Children’s Hospital, 11000 Belgrade, Serbia

**Keywords:** long-gap esophageal atresia, delayed primary anastomosis, Foker procedure, outcomes

## Abstract

**Background/Objectives**: Long-gap esophageal atresia (LGEA) is most commonly associated with Gross types A and B, which are usually diagnosed preoperatively, but may also be encountered in Gross type C, where the long gap is recognized intraoperatively, during fistula ligation. This study aimed to describe and explore outcomes associated with two native esophagus-preserving procedures, delayed primary anastomosis (DPA) and the Foker procedure (FP), and to assess their respective roles in the management of LGEA. **Methods**: We analyzed a population-representative cohort of 38 patients with LGEA treated at two tertiary centers in Belgrade between 2003 and 2023. Patients treated with DPA (*n* = 20) or FP (*n* = 18) were evaluated regarding Gross type, gap length, gastrostomy, treatment duration, complications, major adverse outcomes (including esophageal replacement, redo surgery, or death), and hospital stay. **Results**: Gross types A and B were analyzed together and were more frequently treated with DPA, whereas FP predominated in type C (*p* = 0.001). Sex, gestational age, birth weight, and gap length did not differ between treatment groups. Gastrostomy was performed in all DPA cases and in 50% of FP cases. No statistically significant differences in complication rates or major adverse outcomes were observed between the DPA and FP groups. Hospital stay was significantly longer in the DPA group (*p* < 0.001), although this may be partially influenced by temporal bias related to the later introduction of FP. **Conclusions:** FP may offer practical advantages in type C LGEA, where thoracic access is already required for fistula ligation, whereas DPA may be a suitable approach in types A/B, avoiding an additional thoracic procedure. These findings suggest that anatomical subtype may be an important consideration in treatment selection in LGEA, although validation in larger multicenter prospective studies is warranted.

## 1. Introduction

Long-gap esophageal atresia (LGEA) is a congenital anomaly in which a separation between the proximal and distal esophageal segments precludes primary anastomosis, most commonly characterized by a gap exceeding 2–3 cm or approximately 3–4 vertebral bodies [1,2]. It accounts for approximately 10–15% of EA cases [3]. LGEA most commonly encompasses Gross types A and B, which are typically diagnosed preoperatively. However, LGEA may also be encountered in Gross types C and D, in which the long gap becomes apparent intraoperatively, during tracheoesophageal fistula ligation [4,5]. The inability to perform a primary anastomosis occurs in approximately 5% of type C EA cases; however, the exact prevalence of LGEA in patients with a distal fistula is difficult to ascertain and may be influenced by variations in surgical expertise [5,6]. Current surgical management of LGEA most commonly involves delayed primary anastomosis (DPA), which relies on spontaneous esophageal growth over 2–3 months, or the Foker procedure (FP), in which traction is applied to the esophageal pouches followed by delayed anastomosis after approximately two weeks, resulting in a shorter overall treatment course [7,8,9,10,11,12]. To date, there is no consensus regarding the optimal surgical approach, and decision-making is largely shaped by the surgeon’s experience and preference [2]. This study aimed to describe and explore the outcomes associated with DPA and FP and to assess their respective roles in the contemporary management of LGEA.

## 2. Materials and Methods

### 2.1. Patient Cohort

This retrospective study included patients with LGEA who underwent surgical treatment with DPA or FP between 2003 and 2023 at two national tertiary referral centers in Belgrade, Serbia—University Children’s Hospital (UCH) and Mother and Child Health Care Institute of Serbia “Dr. Vukan Cupic” (MCHCIS)—which collectively manage the vast majority of patients with EA in the country. Clinical data were obtained from archived institutional medical records at both centers. Prenatal suspicion of EA was present in most cases, with the diagnosis confirmed after birth in all patients. Patients treated exclusively with esophageal replacement, without undergoing native esophagus preservation procedures, were excluded. The study was approved by the Ethics Committees of UCH (approval No. 16/26; Date: 15 December 2025) and MCHCIS (approval No. 8/129; Date: 2 November 2025) and was conducted in accordance with the Declaration of Helsinki.

### 2.2. Surgical Techniques

LGEA was defined as any EA in which the distance between the proximal and distal esophageal segments precluded primary anastomosis without excessive tension. The diagnosis of LGEA was presumed preoperatively in patients with Gross types A and B based on the anatomical subtype, whereas in patients with Gross type C, it was established intraoperatively. Gap length was assessed radiographically and expressed as the number of vertebral bodies between the proximal and distal esophageal pouches.

The DPA technique has been used at our institutions since 2003. In all patients treated with DPA, gastrostomy was performed within the first few days of life, followed by several months of watchful waiting. During this period, all patients remained hospitalized until definitive esophageal repair. In patients with Gross type C LGEA, initial ligation of the distal tracheoesophageal fistula was performed through a thoracotomy, whereas in the three patients with Gross type B, the proximal tracheoesophageal fistula was ligated through a cervical approach.

**Figure 1:**
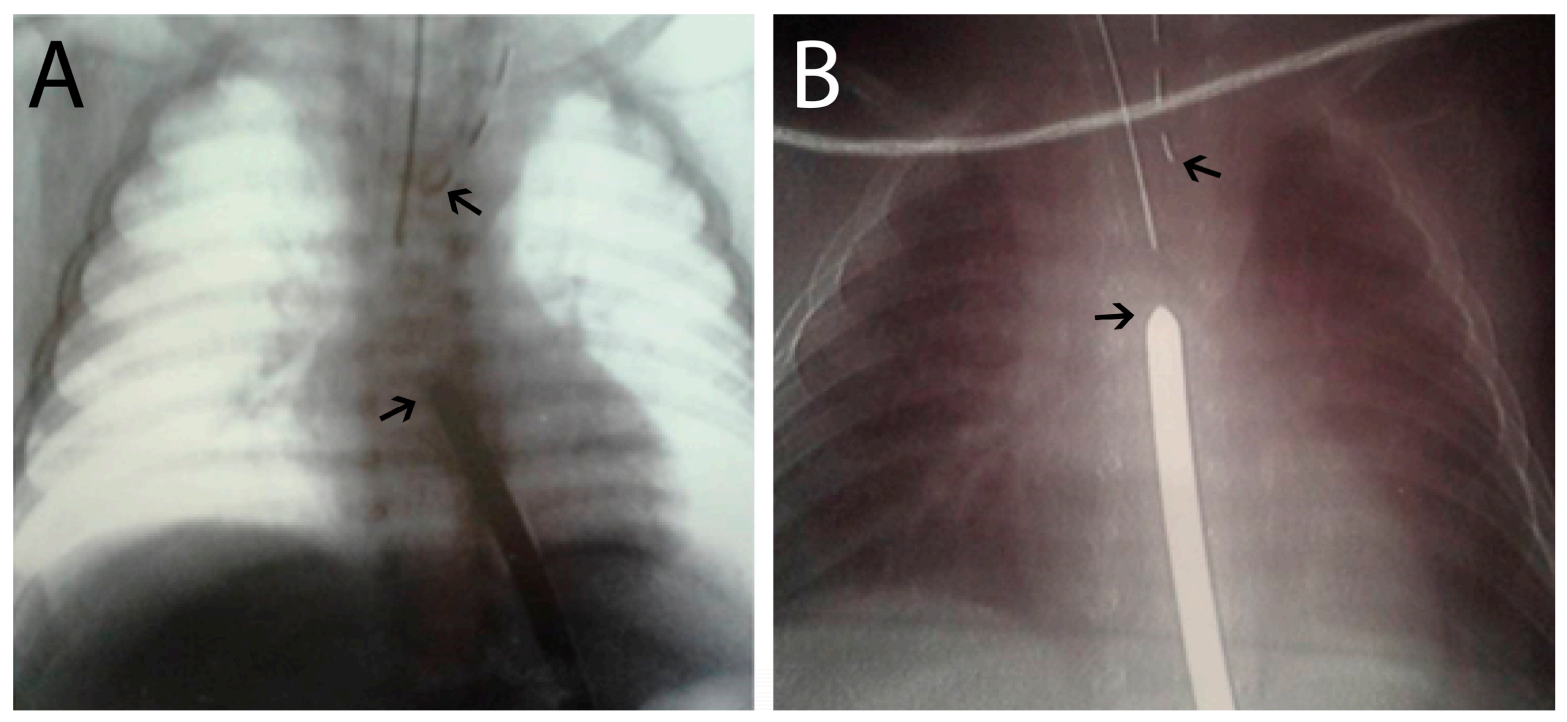
Serial measurements of esophageal gap length before delayed primary anastomosis, showing the initial gap length (**A**) and subsequent reduction in the gap (**B**). Arrowheads indicate the radiopaque tube in the proximal esophageal pouch and the metallic bougie in the distal esophageal segment.

Serial gap assessments were performed every two to three weeks using chest radiographs, with a radiopaque tube placed in the proximal esophageal pouch and a gastric endoscope or metallic bougie introduced through the gastrostomy to delineate the distal esophagus (Figure 1A,B). Definitive DPA was subsequently performed via extrapleural thoracotomy through the fourth intercostal space once sufficient spontaneous reduction in the esophageal gap had occurred.

**Figure 2:**
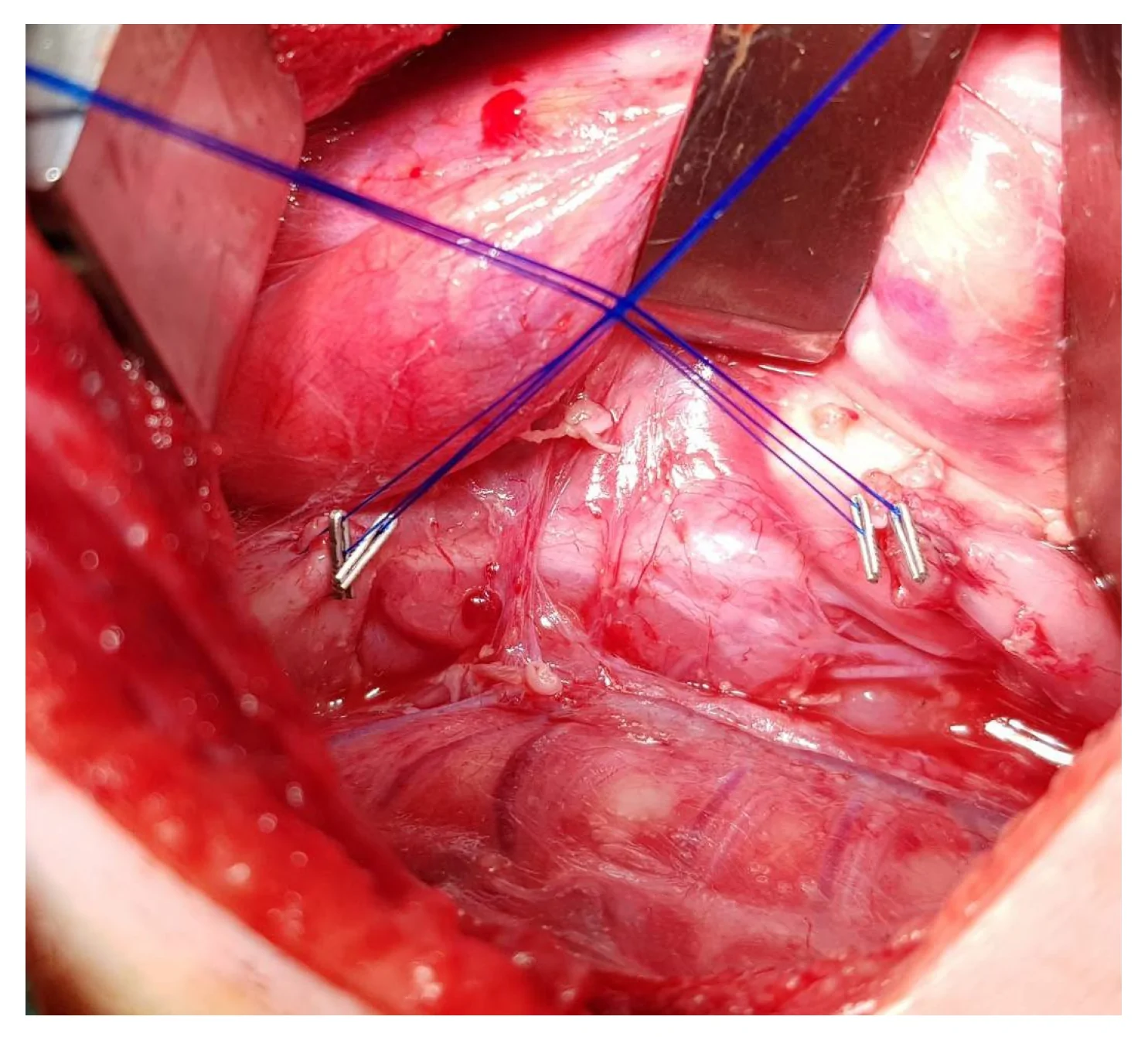
Placement of traction sutures during the Foker procedure.

The FP was adopted at our institutions in 2012 and was performed through a thoracotomy. Following ligation of the tracheoesophageal fistula, when present, the proximal and distal esophageal segments were extensively mobilized. The initial gap length was then assessed. As previously described in the literature [13], external traction was established by placing double purse-string traction sutures at both esophageal ends, which were marked with metallic clips (Figure 2).

**Figure 3:**
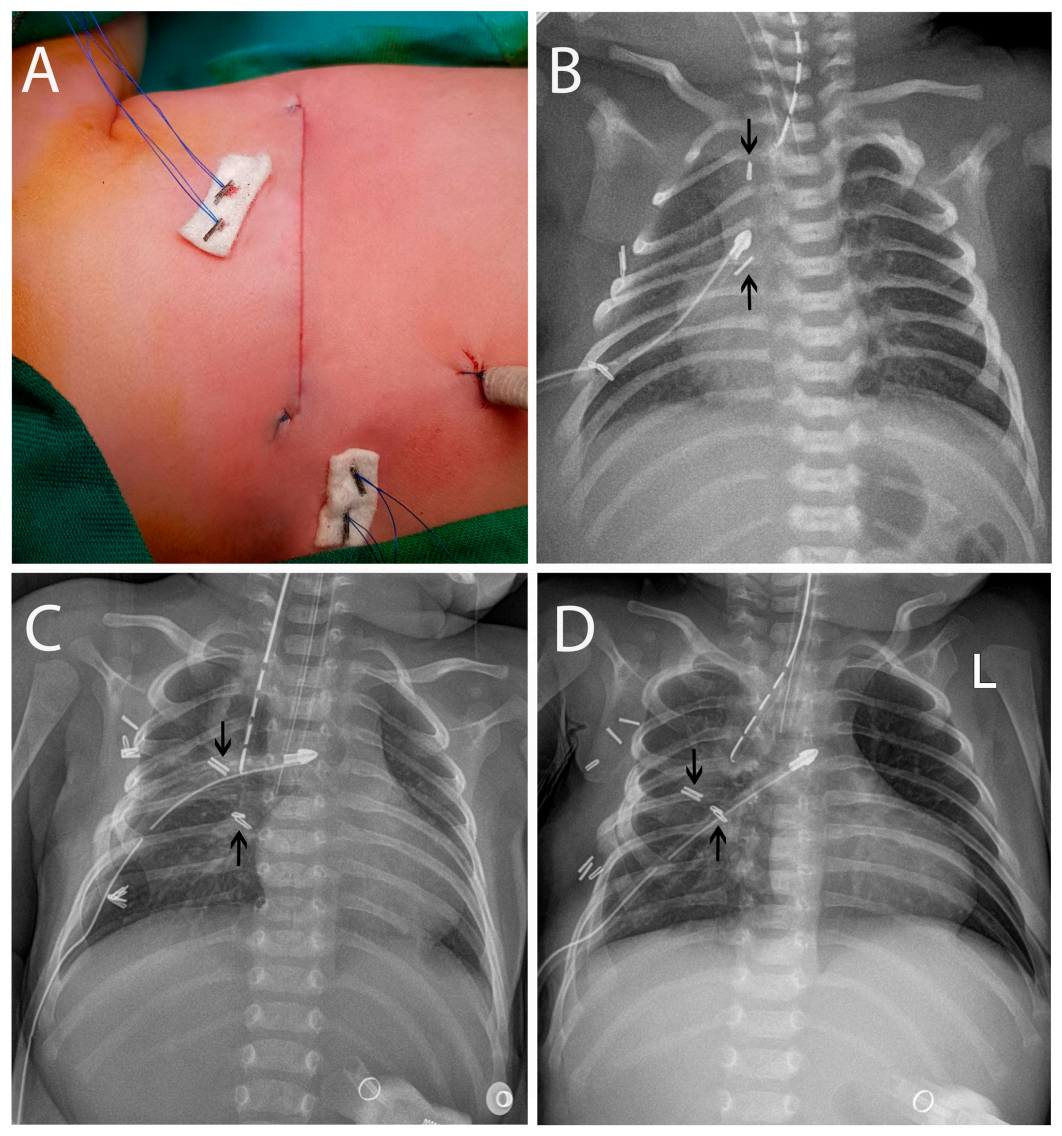
External traction and stepwise reduction in the esophageal gap during the Foker procedure. Traction sutures are externalized through the chest wall, secured to the skin, and periodically retightened (**A**). Serial chest radiographs demonstrate progressive reduction in the esophageal gap (**B**–**D**); arrowheads indicate the metallic clips marking the ends of the proximal and distal esophageal pouches. L, left.

The intensity and duration of traction were adjusted according to intraoperative findings, particularly the elasticity of the esophageal ends. In general, the traction sutures were tightened every 2–3 days at the patient’s bedside. Portable chest radiographs were obtained after placement of the traction sutures and routinely after each subsequent tightening (Figure 3A–D).

Anastomosis was performed once radiographic assessment demonstrated a reduction in the gap to less than one vertebral body or an overlap of the esophageal segments.

In 13 patients, all of whom had Gross type C, traction was initiated within the first three days of life. In the remaining five patients treated with the FP (comprising four patients with Gross type A and one patient with Gross type B), gastrostomy was performed on the first or second day of life, and traction was initiated later (median 32 days, range 16–53 days), following a period of observation during which serial radiographic assessments demonstrated insufficient spontaneous reduction in the esophageal gap.

### 2.3. Clinical Variables and Outcome

Collected variables included sex, gestational age, birth weight, EA Gross type, gap length, gastrostomy, treatment timing and duration, and major complications, namely anastomotic leak, anastomotic stricture, and gastroesophageal reflux (GER). Anastomotic leak was considered present when clinical signs of leakage, including air or salivary drainage through the chest tube, were observed and/or extraluminal leakage of contrast material from the esophageal anastomosis was demonstrated on contrast study. Anastomotic stricture was defined as symptomatic narrowing at the anastomotic site, confirmed by contrast study and/or upper gastrointestinal endoscopy, and requiring dilatation. GER was diagnosed based on clinical symptoms consistent with reflux, supported by findings on contrast study and/or upper gastrointestinal endoscopy. Outcomes were assessed during a 12-month follow-up period, with follow-up completed in 2024. A major adverse outcome was defined as death or the need for redo surgery or esophageal replacement. Patients who did not experience any of these events were classified as having no major adverse outcome, regardless of the occurrence of other complications. Redo surgery referred to any major reoperation related to EA or tracheoesophageal fistula following definitive esophageal repair, excluding endoscopic procedures such as anastomotic dilatation.

### 2.4. Statistical Analysis

Results were presented as frequencies and percentages for categorical variables, and as mean ± standard deviation (SD) or median with interquartile range (IQR) for continuous variables, as appropriate. Differences between groups were assessed using the independent samples *t*-test for normally distributed continuous variables and the Mann–Whitney U test for non-normally distributed continuous variables. Differences between categorical variables were analyzed using the chi-square test or Fisher’s exact test, as appropriate. Effect estimates were reported with 95% confidence intervals (CIs), using odds ratios (ORs) for categorical variables, and mean differences (MDs) or Hodges–Lehmann (HL) estimates for continuous variables, as appropriate. A *p*-value < 0.05 was considered statistically significant. Given the small sample size and limited number of major adverse outcomes, multivariable analysis was not performed, as the available data did not permit reliable adjustment for multiple clinically relevant confounders without substantially increasing the risk of model overfitting. Analyses were conducted using IBM SPSS Statistics version 23.0 (IBM Corp., Armonk, NY, USA) and R software version 4.5.2 (R Foundation for Statistical Computing, Vienna, Austria).

## 3. Results

**Figure 4:**
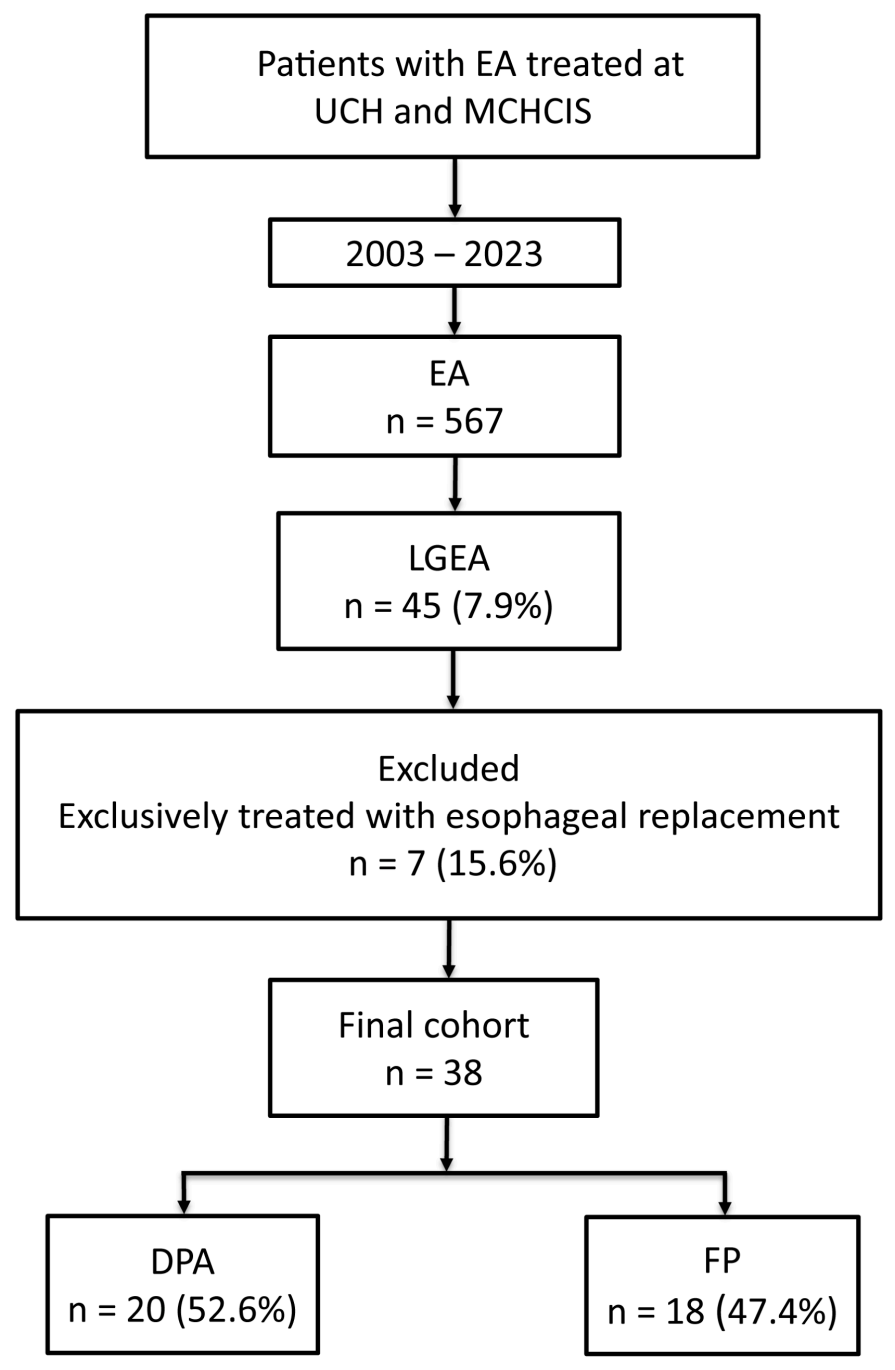
Patient selection flowchart. DPA, delayed primary anastomosis; EA, esophageal atresia; FP, Foker procedure; LGEA, long-gap esophageal atresia; MCHCIS, Mother and Child Health Care Institute of Serbia; UCH, University Children’s Hospital.

During the study period, 567 patients with EA were managed at UCH and MCHCIS. Among them, 45 (7.9%) children were diagnosed with LGEA. Seven patients who underwent exclusively esophageal replacement without attempting native esophagus preservation (six with Gross type A and one with type C EA) were excluded. The final cohort comprised 38 patients, including 20 treated with DPA and 18 who underwent FP (Figure 4). Individual patient-level data are presented in Supplementary Table S1, whereas the baseline characteristics of the study cohort are summarized in Table 1. Complete data were available for all included baseline variables. Due to the small sample size, patients with Gross types A and B were grouped for further statistical analysis. DPA was used significantly more often in patients with Gross types A and B than in those with type C EA, whereas FP was more commonly used in type C EA than in types A and B (80.0% vs. 20.0% and 72.2% vs. 27.8%, respectively; *p* = 0.001).

No significant differences were observed between patients undergoing DPA and FP with respect to sex (*p* = 0.272), gestational age (*p* = 0.535), birth weight (*p* = 0.243), or gap length (*p* = 0.516). Gastrostomy was performed in all patients undergoing DPA and in 50% of those undergoing FP (*p* < 0.001). Hospital stay was significantly longer in patients undergoing DPA than in those undergoing FP (*p* < 0.001) (Table 1).

Complications and outcomes following DPA and FP are summarized in Table 2. The incidence of common post-anastomotic complications, including anastomotic leak, anastomotic stricture, and GER, did not differ significantly between the DPA and FP groups.

During the one-year follow-up, six patients experienced major adverse outcomes. Esophageal replacement was required in two patients, both from the DPA group. In the first patient, after several months of observation, the esophageal ends failed to grow sufficiently to allow a safe primary anastomosis. Thoracotomy was performed in an attempt to mobilize the esophageal ends; however, no additional length could be obtained, and primary anastomosis was therefore considered excessively risky. A cervical esophagostomy was created during the same procedure, followed by delayed esophageal replacement using a colonic conduit. In the second patient, jejunal interposition was performed because of persistent anastomotic stricture refractory to repeated dilatations. In addition, one Gross type C patient from the DPA group required a reoperation for a recurrent tracheoesophageal fistula.

There was one death in the DPA group due to sepsis. In the FP group, two deaths occurred due to complications of associated congenital anomalies. One patient had tetralogy of Fallot, atrial septal defect, patent ductus arteriosus, bilateral hydronephrosis, and anorectal malformation. The second patient had trisomy 21, ventricular septal defect, severe pulmonary hypertension, and multiple limb anomalies. Both patients developed progressive respiratory and hemodynamic deterioration, ultimately leading to multiorgan failure and death.

No significant difference was observed between the DPA and FP groups in the distribution of major adverse outcomes (*p* = 0.663).

## 4. Discussion

EA in Serbia is treated at three referral centers, with UCH and MCHCIS managing the vast majority of patients. This centralized referral pattern supports the population-representative nature of our cohort. Although the cohort size is relatively small, reflecting the rarity of LGEA, the inclusion of 38 patients exceeds the sample sizes of many previously published institutional series on LGEA [4,6,7,14,15,16,17,18] and provides a population-representative overview of LGEA management within a national two-center setting.

LGEA remains a major challenge in pediatric surgery, particularly when preservation and reconstruction of the native esophagus are pursued, as this approach is associated with better long-term functional outcomes than esophageal replacement procedures [1,8,15,19]. Moreover, esophageal replacement procedures are technically demanding and require additional specialized surgical expertise [1,19,20]. In our population-representative cohort, LGEA accounted for 7.9% of all EA cases, which is slightly lower than previously reported in the literature [1,2,8]. Although LGEA is most commonly associated with Gross type A, the term “long-gap” does not define or depend on a specific anatomical subtype and should not be considered interchangeable with Gross type A EA [1]. For practical purposes, LGEA may be defined as a condition in which the gap between the esophageal ends is too wide to permit a secure primary anastomosis without excessive tension [8,10,15,18,21]. This definition was also used in our cohort to guide patient management.

To date, no single surgical technique has emerged as clearly superior for the treatment of LGEA [19,22]. Therefore, management remains highly dependent on institutional experience and available surgical expertise [23]. Allowing spontaneous esophageal growth without using invasive techniques is generally preferred when feasible. This process is most pronounced during the first 8–12 weeks of postnatal development, and DPA becomes possible once the distance between the proximal and distal esophageal segments has decreased to less than 2 cm [12]. Accordingly, DPA is widely advocated as the initial treatment strategy for LGEA and is supported by the recommendations of the American Pediatric Surgical Association (APSA) and the 2019 European Reference Network for Rare Inherited Congenital Anomalies (ERNICA) consensus conference [1,23].

The survival rate of patients treated with DPA in our study was 95%, consistent with previously reported data [12]. According to several studies, anastomotic leakage occurs in approximately 30% of patients treated with DPA; however, most leaks are minor and resolve with conservative management [12,24]. This rate is comparable to that observed in our cohort (35%). Literature data indicate that anastomotic strictures occur in up to 60% of patients treated with DPA, with the majority responding well to periodic dilatation, although some may be refractory, particularly in the presence of concomitant GER [12,24]. Among patients treated with DPA in our study, postoperative esophageal stricture occurred less frequently, affecting 40% of patients. Many series have reported the need for fundoplication in up to 30% of patients within the first year after DPA [12,24], whereas fundoplication was required in only 10% of our patients. The principal limitations of DPA remain prolonged hospitalization and the ongoing risk of aspiration pneumonia before definitive repair. In our series, the mean duration of hospitalization was 167.4 ± 55.2 days, reflecting the extended interval required to achieve esophageal continuity, with a median time to anastomosis of five months (range, 1.5–7 months). Published series have reported substantial variability in the timing of DPA, encompassing both shorter and longer intervals than those observed in our cohort [25,26].

In patients who demonstrate insufficient spontaneous esophageal growth or present with extremely long gaps, traction-based techniques have been developed to accelerate esophageal elongation and shorten the time to definitive repair. The FP, which utilizes external traction sutures, allows rapid elongation of the esophageal segments and often enables anastomosis within two weeks [1,9,16,27]. The classic open FP is invasive and has historically required at least two thoracotomies—one for traction suture placement and another for definitive anastomosis. Reported complications include esophageal perforation and mediastinitis during traction-induced elongation, as well as anastomotic leak, anastomotic stricture, and GER following anastomosis [27,28]. However, outcomes appear to be strongly influenced by institutional and surgeon experience, with high-volume centers reporting improved safety profiles and more reproducible results [1,9].

In our cohort, no adverse events related to traction sutures, such as esophageal tearing or mediastinitis, were observed in patients treated with the FP. While earlier reports documented traction-related esophageal leaks in up to 22% of patients, contemporary series from experienced centers report rates as low as 4%, reflecting substantial improvements associated with increasing experience and technical refinements [9,16]. In addition to meticulous tissue handling and careful adjustment of traction throughout the elongation process, double purse-string traction sutures routinely used on both esophageal segments in our series may also be relevant when considering the absence of traction-related complications.

Studies from major FP centers have reported anastomotic leak rates ranging from 12% to 37%, reflecting both differences in patient populations and progressive refinements in technique over time [9,28]. In our cohort, anastomotic leak occurred in 16.7% of FP patients. Anastomotic stricture remains one of the most common complications following the FP and affects most patients treated with this technique, according to contemporary series [9]. This complication occurred in 55.6% of our patients who underwent the FP. GER was documented in 66.7% of FP patients in our series, although most cases were managed conservatively. Severe reflux requiring fundoplication has been reported in 39–67% of patients in large FP series [9,28], compared with 27.8% in our cohort. Lower rates of these complications have also been reported in a series employing delayed traction after a period of spontaneous esophageal growth, highlighting the potential impact of reduced tension, esophageal maturation, and less extensive dissection on postoperative outcomes [7]. Finally, the two deaths observed among FP-treated patients in our study were not attributable to the esophageal surgery.

The duration of hospitalization was significantly longer in the DPA group than in the FP group. Although this difference is likely primarily attributable to the longer time to anastomosis in DPA patients, the potential influence of the different study periods cannot be excluded. The cohort included patients treated from 2003 onward, whereas the FP was introduced in 2012. Advances in neonatal intensive care, anesthesia, and perioperative management over this period might therefore have contributed to the shorter hospital stay observed in the FP group, making it difficult to attribute this difference solely to the surgical strategy.

During the one-year follow-up in our study, no statistically significant differences were observed in overall outcomes or complication rates between patients treated with DPA and those treated with FP. However, the wide confidence intervals around the effect estimates indicate considerable uncertainty and limit the precision of these comparisons. Additionally, these findings should be interpreted in the context of the marked differences in anatomical subtype between the groups. Our data revealed a distinct pattern in surgical decision-making, with Gross types A and B predominantly treated with DPA or, in selected cases, with later initiation of FP, whereas early FP traction was more frequently employed in patients with Gross type C. This distribution likely reflects deliberate procedure selection based on anatomical subtype and clinical considerations rather than a predefined institutional protocol. Moreover, the timing of traction initiation in patients treated with the FP reflected evolving clinical practice over the extended study period. The learning curve following the introduction of the FP in 2012 might also have influenced complication rates and other outcomes. Consequently, the observed outcomes reflect not only the surgical strategy but also underlying anatomical differences and treatment selection and should therefore not be interpreted as direct comparative effects of the two techniques. Nevertheless, taken together, these observations suggest an individualized approach to LGEA management, in which surgical strategy may be guided by anatomical subtype and practical clinical considerations.

In long-gap type C EA, the presence of a distal tracheoesophageal fistula necessitates early surgical intervention to prevent respiratory complications. Access to the mediastinum via thoracotomy or thoracoscopy is required shortly after birth to ligate the fistula. We propose that this obligatory intervention represents a unique “window of opportunity.” Rather than performing fistula ligation alone followed by prolonged waiting for spontaneous growth, this initial procedure can also be used to place traction sutures, thereby transforming a protective maneuver into an active step toward esophageal elongation [4]. Using FP as the primary strategy in type C LGEA allows surgeons to capitalize on the required thoracic access and may shorten the time to definitive repair, thereby decreasing the need for gastrostomy and enabling earlier initiation of oral feeding. Prolonged absence of oral feeding and extended dependence on gastrostomy have been associated with feeding aversion and impaired oral–motor development. Earlier restoration of esophageal continuity may therefore offer functional benefits beyond anatomical repair [29]. However, the need for gastrostomy should not be interpreted solely as a complication but also as an indicator of disease severity and the chosen treatment strategy.

In contrast, the management rationale differs in Gross type A LGEA, where no tracheoesophageal fistula is present. There is no urgent indication for thoracic exploration in the neonatal period, and performing an open FP would require a thoracotomy solely for traction suture placement, representing a substantial surgical burden. DPA may therefore represent a suitable treatment strategy in Gross type A atresia, allowing spontaneous esophageal growth and limiting surgical trauma to a single definitive thoracotomy once the gap has sufficiently decreased. In this context, DPA adheres to the principle of minimal invasiveness, reserving traction-based techniques for cases in which spontaneous growth ceases or proves inadequate. A similar strategy may also be appropriate in selected patients with Gross type B EA, as the proximal tracheoesophageal fistula can often be ligated through a cervical approach [30,31], thereby avoiding additional thoracotomy.

The development of thoracoscopic techniques may modify LGEA treatment approaches in the future. Thoracoscopy can be applied across several treatment strategies for LGEA, including DPA, placement of external traction sutures, and completely minimally invasive internal traction techniques [32]. Although thoracoscopic traction techniques have been increasingly reported and may reduce surgical trauma compared with traditional open techniques, they remain technically demanding and are associated with a steep learning curve. Consequently, their application is currently limited to highly specialized centers, precluding widespread adoption [14,21]. Even in highly developed countries such as the United States and Germany, only a minority of EA patients have been managed thoracoscopically [33,34]. Nevertheless, several specialized European centers have adopted thoracoscopy as the standard approach for nearly all EA patients, and the number of centers performing thoracoscopic EA repair continues to grow worldwide [35,36,37]. Finally, thoracoscopic treatment of LGEA remains particularly challenging and is associated with substantial complication rates. Consequently, these patients should be managed in specialized referral centers by experienced surgeons within dedicated multidisciplinary teams [38].

This study has several limitations. Its retrospective design, relatively small sample size, limited number of major adverse events, and non-randomized treatment allocation limit the statistical power and preclude definitive conclusions regarding the relative effectiveness of the two surgical strategies. In particular, the marked differences in anatomical subtype between the groups introduce confounding by indication, and the absence of statistically significant differences should not be interpreted as evidence of equivalence. The extended study period also introduces potential temporal bias due to evolving clinical practice and perioperative care. In addition, complete data on associated congenital anomalies were not available for all patients. Finally, the relatively short follow-up period precluded assessment of important long-term outcomes, including swallowing function, feeding ability, nutritional status, quality of life, the need for repeated esophageal dilatations, and persistent or recurrent GER. Nevertheless, the population-representative nature of the cohort provides a valuable real-world perspective on contemporary LGEA management and reflects treatment practices within a national referral setting.

## 5. Conclusions

Both DPA and FP were successfully used to achieve continuity of the native esophagus in our cohort of LGEA patients. Our findings suggest that anatomical subtype may be an important consideration in surgical decision-making. DPA may be a suitable approach for Gross type A and selected patients with Gross type B EA, avoiding an additional thoracic procedure while allowing spontaneous esophageal growth. In contrast, FP may offer practical advantages in patients with type C LGEA, where thoracic access is already required for fistula ligation and can also be used to initiate traction. Accordingly, an individualized, anatomy-driven treatment approach may help minimize surgical burden in this challenging patient population. Such a pragmatic real-world approach may remain particularly relevant during the ongoing transition toward broader implementation of advanced thoracoscopic techniques, which are likely to become increasingly integrated into routine clinical practice in the future. However, given the retrospective, non-randomized design and limited sample size, these findings should be considered hypothesis-generating and require validation in larger multicenter prospective studies before definitive treatment recommendations can be made.

## Figures and Tables

**Table 1:** Baseline clinical characteristics of patients with LGEA according to surgical approach (DPA vs. FP).

	DPA (*n* = 20)	FP (*n* = 18)	Effect Estimate (95% CI)	*p*-Value
EA type				
A	13 (65.0%)	4 (22.2%)		
B	3 (15.0%)	1 (5.6%)		
C	4 (20.0%)	13 (72.2%)		
EA type—grouped				
A + B	16 (80.0%)	5 (27.8%)	OR 10.40 (2.31 to 46.83)	**0.001** ^a^
C	4 (20.0%)	13 (72.2%)		
Sex				
Male	11 (55.0%)	13 (72.2%)	OR 0.47 (0.12 to 1.83)	0.272 ^a^
Female	9 (45.0%)	5 (27.8%)		
Gestational age, weeks (mean ± SD)	35.6 ± 2.2	36.1 ± 2.1	MD −0.50 (−1.92 to 0.92)	0.535 ^c^
Birth weight, g (mean ± SD)	2199.5 ± 540.7	2396.1 ± 472.4	MD −196.6 (−530.0 to 136.8)	0.243 ^c^
Gap length, VB (median, IQR)	4.7 (4.0–5.0)	4.0 (3.9–5.2)	HL 0.00 (−0.50 to 1.00)	0.516 ^d^
Gastrostomy				
Yes	20 (100.0%)	9 (50.0%)		<**0.001** ^b^
No	0	9 (50.0%)		
Time to DPA, months (median, IQR)	5 (4.0–6.0)	—		
Time to initial FP traction, days (median, IQR)	—	2.0 (1.0–19.7)		
Traction duration, days (mean ± SD)	—	14.7 ± 3.9		
Total time to anastomosis in FP patients, days (median, IQR)	—	19.5 (14.0–31.5)		
Hospital stay, days (mean ± SD)	167.4 ± 55.2	62.2 ± 16.0	MD 105.2 (78.5 to 131.9)	**<0.001** ^c^

Data are presented as *n* (%), mean ± SD, or median (IQR), as appropriate. *p*-values were calculated using the χ^2^ test (^a^) or Fisher’s exact test (^b^) for categorical variables and Student’s *t*-test (^c^) or Mann–Whitney U test (^d^) for continuous variables, as appropriate. For ORs, the FP group was used as the reference category. MD and HL estimates were calculated in the direction DPA − FP. Bold values indicate statistical significance (*p* < 0.05). CI, confidence interval; DPA, delayed primary anastomosis; EA, esophageal atresia; FP, Foker procedure; HL, Hodges–Lehmann estimate; IQR, interquartile range; MD, mean difference; OR, odds ratio; SD, standard deviation; VB, vertebral bodies.

**Table 2:** Complications and outcomes of patients treated with DPA and FP.

	DPA (*n* = 20)	FP (*n* = 18)	OR (95% CI)	*p*-Value
Complications				
Anastomotic leak	7 (35.0%)	3 (16.7%)	2.69 (0.58 to 12.60)	0.278 ^a^
Anastomotic stricture	8 (40.0%)	10 (55.6%)	0.53 (0.15 to 1.94)	0.338 ^b^
GER (total)	10 (50.0%)	12 (66.7%)	0.50 (0.13 to 1.86)	0.299 ^b^
GER (conservative)	8 (40.0%)	7 (38.9%)	1.05 (0.28 to 3.86)	0.944 ^b^
GER (surgical)	2 (10.0%)	5 (27.8%)	0.29 (0.05 to 1.73)	0.222 ^a^
Major adverse outcome				
No	16 (80.0%)	16 (88.9%)		0.663 ^a^
Yes	4 (20.0%)	2 (11.1%)	2.00 (0.32 to 12.51)	
Major adverse outcomes				
Esophageal replacement	2 (10.0%)	0 (0.0%)		
Redo surgery	1 (5.0%)	0 (0.0%)		
Death	1 (5.0%)	2 (11.1%)		

*p*-values were calculated using Fisher’s exact test (^a^) or the χ^2^ test (^b^), as appropriate. For ORs, the FP group was used as the reference category. CI, confidence interval; DPA, delayed primary anastomosis; FP, Foker procedure; GER, gastroesophageal reflux; OR, odds ratio.

## Data Availability

The original contributions presented in this study are included in the article/Supplementary Materials. Further inquiries can be directed to the corresponding author(s).

## Supplementary Materials

The following supporting information can be downloaded at: https://www.mdpi.com/article/10.3390/jcm15176895/s1, Supplementary Table S1: Individual patient-level data, including clinical characteristics, treatment details, complications, and outcomes in children with LGEA.
