# Supplementary material for: Surgical Strategies for Native Esophagus Preservation in Long-Gap Esophageal Atresia: A 20-Year Population-Based Study
Source: J Clin Med. 2026 Sep 6;15(17):6895. doi: 10.3390/jcm15176895 (PMC13565888; doi:10.3390/jcm15176895)
Supplement: Supplementary file 1 [file jcm-15-06895-s001.zip › jcm-4473718-supplementary.pdf]

Supplementary Table S1. Individual patient-level data, including clinical characteristics, treatment details, complications, and outcomes in children with LGEA

Patient Number	Surgical Procedure	Sex	Gestational age (weeks)	Birth weight (g)	Gross type	Gastrostomy	Gap Length (vertebral bodies)	Time to DPA (months)	Time to initial FP traction (days)	Traction duration (days)	Total time to anastomosis in FP (days)	Complications						Outcome	Hospital stay (days)
												Leak	Stricture	Rec. TEF	Insufficient esophageal growth	GER (conservative)	GER (surgical)		
1	DPA	m	34	1950	A	+	4	6										favorable	207
2	DPA	m	35	2100	A	+	6	7				+	+			+	+	favorable	243
3	DPA	f	36	2780	A	+	4	1.5				+	+			+		JI	92
4	DPA	m	35/36	2200	A	+	5	1.5										favorable	67
5	DPA	m	37	2800	A	+	3	1.5					+			+		favorable	44
6	DPA	m	34	1800	A	+	4	5				+						death	180
7	DPA	m	34	1640	A	+	6	6				+	+					favorable	209
8	DPA	m	35	1980	A	+	5	4.5					+			+		favorable	152
9	DPA	f	35	2200	A	+	4.5	5								+	+	favorable	178
10	DPA	f	37	2750	A	+	5	6										favorable	201
11	DPA	f	36	2150	A	+	4	4										favorable	154
12	DPA	f	38	2600	A	+	4	4										favorable	150
13	DPA	f	37	2360	A	+	5	5								+		favorable	180
14	DPA	m	36	2030	B	+	5	5				+	+			+		favorable	177
15	DPA	f	40	3100	B	+	4	4										favorable	149
16	DPA	f	36	2120	B	+	6	6				+	+			+		favorable	208
17	DPA	m	30	880	C	+	3	3.5						+	+			redo	149
18	DPA	m	37	2540	C	+	5	4.5										favorable	153
19	DPA	f	38	2710	C	+	4	5				+	+					favorable	179
20	DPA	m	32/33	1300	C	+	6	7										CI	271
21	FP	m	38/39	2400	A	+	3.5		32	15	47	+				+	+	favorable	70
22	FP	f	37	2400	A	+	5		44	10	54					+		favorable	77
23	FP	m	39	2300	A	+	4.5		53	13	66		+			+		favorable	62
24	FP	m	35/36	2180	A	+	4		16	12	28							favorable	62
25	FP	f	35	2200	B	+	6		31	11	42		+			+	+	favorable	77
26	FP	m	34/35	2640	C	-	4		2	11	13	+	+			+	+	favorable	85
27	FP	m	36	3610	C	-	3		2	16	18							favorable	40
28	FP	m	34	1700	C	+	4		1	22	23		+			+	+	favorable	73
29	FP	m	39	2310	C	-	6		3	21	24		+					death	53
30	FP	m	38/39	3100	C	-	4		3	14	17		+					death	77
31	FP	f	37	2100	C	-	5		1	17	18		+					favorable	38
32	FP	f	36	2730	C	+	6		1	12	13							favorable	33
33	FP	m	37	2360	C	-	4		1	13	14					+	+	favorable	65
34	FP	m	34	2060	C	-	5		2	12	14							favorable	61
35	FP	f	33	1900	C	+	6		3	17	20		+					favorable	62
36	FP	m	32	1740	C	-	3		1	10	11							favorable	75
37	FP	m	35/36	2750	C	-	3		1	18	19							favorable	40
38	FP	m	38	2650	C	+	4		2	21	23	+	+					favorable	70

CI colonic interposition; DPA, delayed primary anastomosis; EA, esophageal atresia; FP, Foker procedure; GER, gastroesophageal reflux; JI, jejunal interposition; Rec. TEF, recurrent tracheoesophageal fistula.
